# “Avoir la peau claire ……et pourquoi pas? ”: dépigmentation volontaire chez les femmes dans une région du sud-ouest du Bénin

**DOI:** 10.11604/pamj.2019.33.72.18722

**Published:** 2019-05-31

**Authors:** Yolaine Glèlè-Ahanhanzo, Alphonse Kpozehouen, Boniface Maronko, Colette Azandjèmè, Virginie Mongbo, Charles Sossa-Jérôme

**Affiliations:** 1 Département d'Epidémiologie et de Biostatistiques, Institut Régional de Santé Publique, Université d'Abomey-Calavi, Bénin; 2 Département de Promotion de la Santé, Institut Régional de Santé Publique, Université d'Abomey-Calavi, Bénin; 3 Département Politiques et Systèmes de Santé, Institut Régional de Santé Publique, Université d'Abomey-Calavi, Bénin

**Keywords:** Agents éclaircissants pour la peau, santé des femmes, facteurs de risque, Bénin, Survival, prognostic factor, esophageal cancer, Cameroon

## Abstract

**Introduction:**

Cette étude avait pour objectif de déterminer la prévalence et les facteurs associés à l'usage des produits de dépigmentation volontaire chez les femmes de 15 à 49 ans dans la zone sanitaire de Comè au Bénin.

**Méthodes:**

Il s'agit d'une étude transversale réalisée en 2016 et qui a concerné 511 femmes âgées de 15 à 49 ans sélectionnées par sondage en grappes. Les données ont été collectées par questionnaire avec des informations sur les caractéristiques démographiques, socio-culturels et économiques des femmes. Les produits de dépigmentation ont été identifiés dans la liste de composition des laits corporels déclarés d'utilisation régulière. L'analyse s'est basée sur une régression logistique multiple. Le seuil statistique de signification a été fixé à 5%.

**Résultats:**

La prévalence de l'usage des produits de dépigmentation volontaire chez les femmes de 15 à 49 ans dans la zone sanitaire de Comè était de 79,22% IC95%=[75,72-82,78] et 84,23% des femmes connaissaient au moins un des effets néfastes sur la santé liés à l'usage des produits de dépigmentation cutanée. Les produits utilisés étaient à base d'hydroquinone à 98,24% et des dermocorticoïdes à 1,76%. Les facteurs associés à l'usage des produits de dépigmentation volontaire étaient l'état matrimonial (célibataire, veuve, séparée ou divorcée) de l'enquêtée (OR =3,1; IC95%=[1,29-7,44]), la recherche d'un mari ou de partenaire (OR=4,92; IC 95%=[1,20-20,09]), l'existence des taches hyperpigmentées (OR=10,32; IC95%=[2,87-37,01]).

**Conclusion:**

Ces résultats ont montré que l'usage des produits de dépigmentation cutanée chez les femmes est un problème préoccupant de santé publique et de grande ampleur dans la zone sanitaire Comè; des interventions intégrées de communication pour un changement de comportement doivent être entreprises.

## Abstract (en)

**Introduction:**

This study aims to determine the prevalence and the factors associated with the use of products for voluntary depigmentation among women aged 15-49 years in Comè health zone in Benin.

**Methods:**

We conducted a cross-sectional study of 511 women aged 15-49 years selected using a cluster survey in 2016. Data were collected using a questionnaire seeking to explore the demographic, socio-cultural and economic characteristics of women. Depigmentation ingredients have been identified looking at the composition of regularly used body lotions. We performed a multiple logistic regression analysis. The statistical significance level was set at 5%.

**Results:**

The prevalence of use for voluntary depigmentation products among women aged 15-49 years in Comè health zone was 79,22% 95%; CI=[75,72-82,78] and 84,23% of women knew at least one of the adverse health effects associated with the use of skin depigmentation products. The products used were hydroquinone based (98.24%) and dermocorticoid based (1.76%). Factors associated with the use of voluntary depigmentation products included marital status (single, widowed, separated or divorced) (OR=3.1; 95% CI=[1.29-7.44]), the search for a husband or a partner (OR=4.92; 95% CI=[1.20-20.09]), the presence of hyperpigmented spots (OR=10.32; 95% CI=[2.87-37,01]).

**Conclusion:**

These results show that the use of skin depigmentation products among women is a serious and huge public health problem in Comè health zone. Integrated communication campaign which can produce positive changes should be implemented.

## Introduction

Selon la croyance populaire, les femmes qui ont la peau claire sont attrayantes [1]; dans certaines communautés indiennes ou asiatiques, les gens pensent que les chances de se marier et d'appartenir à une caste supérieure, avec les avantages économiques sociaux qui en découlent, sont renforcées par une peau claire [1,2]. L'usage des produits de dépigmentation volontaire (UPDV) est un problème de santé publique [3,4]. Les données sur l'ampleur de la dépigmentation volontaire dans la population générale, sont rares, que ce soit en Afrique, ou dans le monde. Tous les continents sont concernés par le phénomène d'UPDV que ce soit au Moyen-Orient, en Afrique, dans les Caraïbes, en Asie, en Europe, Amérique du Nord ou en Amérique latine [5]. Dans les pays d'Asie, 40 à 59% des femmes utilisent les produits de dépigmentation volontaire [2,6,7]. En Afrique sub-saharienne, 25 à 96% des femmes utilisent les produits de dépigmentation volontaire [5,8-10]. La dépigmentation volontaire est une pratique surtout féminine en Afrique [3,4,10,11]. L'utilisation des produits de blanchiment de la peau peut provoquer des complications systémiques, tels que l'hypertension, le diabète, l'insuffisance surrénalienne, la néphropathie membraneuse [12,13], le syndrome néphrotique [14-16], l'insomnie et la perte de mémoire [17]. Au Bénin, les données brutes sur l'UPDV dans la population générale sont rares. Quelques études ont été réalisées sur le phénomène d'UPDV. Une étude réalisée à Parakou en région urbaine dans le nord du pays, avait montré que la prévalence chez les femmes utilisant les produits de dépigmentation volontaire était très élevée (78,32%) [18]. Les services de santé, après des interventions en santé pour le changement de comportement sur les méfaits de l'utilisation des produits de dépigmentation volontaire, ne communiquent souvent pas sur les résultats de leur intervention. On note des panneaux publicitaires qui prônent pour l'utilisation des “produits éclaircissants”, présents sur tous les grands axes routiers. Les publicités sont également faites sur l'utilisation des produits dépigmentant sur les chaines de télévision. Il n'y a pas de mécanismes mis en place pour contrôler ces publicités contre l'UPDV [3]. Il n'existe pas non plus de réglementation nationale pour lutter contre la fabrication et la vente des produits de dépigmentation. La zone sanitaire de Comè, zone en pleine expansion démographique, zone touristique et frontalière avec la capitale togolaise Lomé, où l'usage de la dépigmentation volontaire est relativement fréquente (59% de cette population enquêtée utilisait les produits de dépigmentation volontaire) [5,19]. Le phénomène de l'UPDV apparait comme une réalité dans cette zone touchant notamment et surtout les femmes en âge de procréer, réalité souvent accompagnée de plusieurs complications individuelles, familiales et communautaires [20]. Afin de proposer des interventions adaptées, il est essentiel de connaitre l'ampleur du phénomène et d'en identifier les éléments explicatifs. Ainsi, l'objectif de la présente étude est d'identifier les facteurs associés à l'usage des produits de dépigmentation volontaire chez les femmes de 15 à 49 ans de la zone sanitaire Comè.

## Méthodes

**Cadre de l'étude**: la zone sanitaire de Comè, située dans le Département de Mono au Sud-ouest de la République du Bénin, pays côtier de la région ouest africaine. Elle est constituée de 4 communes: Bopa, Comé, Grand-Popo et Houéyogbé, de 25 arrondissements et de 200 villages ou quartiers et couvre une superficie de 1120 Km² avec une population en 2016 est estimée à 383 248 habitants dont 85 579 femmes en âge de procréer.

**Type d'étude**: il s'agit d'une étude transversale qui a été réalisée en juillet 2016.

**Echantillonnage**: la population cible de l'étude est constituée par les femmes en âge de procréer, soit âgées de 15 à 49 ans. La taille de l'échantillon a été calculée selon la formule de Schwartz avec une prévalence de 73,62% des femmes pratiquant la dépigmentation volontaire à Parakou en 2011 [18].

$$n=\frac{Z^{2}\alpha *p*q}{i^{2}}$$

avec p=0,7362; q=1-0,7362=0,2638; Le risque d'erreur consenti α=0,05; L'écart réduit au risque consenti: Z_α_=1,96; i=Précision souhaitée pour nos résultats=0,05; g=Effet grappe; ici g=1,5. n=(1,5*(1,96)^2^*0,7362*2,2638)/0,05^2^=449

Tenant compte d'un taux de non réponse de 10%, la taille minimale estimée de l'échantillon est de 494 femmes. Les cibles ont été sélectionnées par technique de sondage en grappes à partir de la liste des 200 villages ou quartiers de ville des quatre communes de Comè, Bopa, Grand Popo et Houéyogbé; 64 grappes ont été retenues de façon aléatoire. Ont été incluses les cibles vivant dans la zone depuis au moins 6 mois.

**Technique, outils de collecte et variables**: un entretien structuré basé sur un questionnaire a permis de recueillir les informations relatives aux différentes variables. La variable principale était l'usage des produits de dépigmentation volontaire". Cette variable était dichotomique: l'usage des produits de dépigmentation"=« Oui » si la femme utilise régulièrement un produit contenant au moins un actif de dépigmentation et “Non”, sinon. Les variables explicatives étaient regroupées selon les caractéristiques socio-démographiques, économiques et l'état de santé de la femme.

**Traitement et analyse des données**: les données ont été saisies à l'aide du logiciel EPIINFO version 7 après vérification de chaque fiche; elles ont été ensuite analysées à l'aide du logiciel STATA 12. Pour tenir compte de la disparité de la population cible dans les différentes grappes, toutes les analyses ont été pondérées. En analyse descriptive, les proportions ou les pourcentages pondérés ont été déterminés pour les variables qualitatives; la moyenne pondérée et l'écart type pour les variables quantitatives dont la distribution était normale. La médiane et l'intervalle interquartile (Q1, Q3) étaient calculés pour les variables quantitatives dont la distribution n'était pas normale. En analyse uni-variée, la recherche d'association entre l'UPDV et les différents facteurs a été faite à l'aide du test statistique Chi2 de Pearson. Les associations entre la variable dépendante et les variables indépendantes ont été appréciées à l'aide des Odds Ratio (OR) et leur intervalle de confiance à 95% (IC95%). En analyse multi-variée, la régression logistique a été utilisée pour identifier les variables associées à l'usage des produits de dépigmentation. Nous avons introduit dans les différents modèles initiaux toutes les variables indépendantes dont le degré de signification était inférieur ou égal à 20% à l'analyse uni-variée. Une stratégie de modélisation à pas à pas descendant a été effectuée pour déterminer les variables statistiquement associées à l'UPDV et à la fin un modèle final a été retenu. Le seuil de signification retenu était de 5%. Le test de Hosmer-Lemeshow a été utilisé pour vérifier l'adéquation du modèle final.

**Considérations éthiques et déontologiques**: nous avons obtenu l'accord des autorités administratives de la zone sanitaire et un consentement éclairé des femmes retenues a été signé, après information des cibles et avant l'administration du questionnaire.

## Résultats

Durant l'enquête, 511 femmes ont été enquêtées.

**Les caractéristiques de la population étudiée**: l'âge médian des femmes enquêtées était de 26 ans [20;34] ans. La tranche d'âge la plus représentée était celle des femmes ayant entre 15 à 24 ans (41,33%). Dans la population de femmes enquêtées, l'ethnie Sahouè était la plus représentée (52,45%), et 62,46% de la population enquêtée pratiquaient la religion chrétienne. Pour leur niveau d'instruction, 29,79% des femmes avaient le niveau primaire, 33,64% le niveau secondaire et 31,57% étaient non scolarisées. Parmi les femmes enquêtées, trois sur huit étaient des commerçantes ou des vendeuses (38,24%), elles vivaient majoritairement en milieu rural (57,71%) et deux femmes sur sept étaient célibataires (29,08%).

**Prévalence de l'UPDV**: la prévalence de l'UPDV chez ces femmes était de 79,25% (IC95%=[75,72-82,78]). Les produits de dépigmentation volontaire étaient à base d'hydroquinone dans 76,71% des cas et de dermocorticoïde dans 2,54%. Les femmes qui faisaient usage de ces produits, le faisaient majoritairement au moins une fois par jour (71,67%). La fréquence d'application d'usage de deux fois par jour était retrouvée dans 61,11% des cas. Les motivations d'usage de ces produits étaient la recherche de la beauté (60,06%), l'estime de soi (34,06%) et la recherche de conjoints ou de partenaires (5,88%); 84,23% des femmes connaissaient au moins un des effets néfastes sur la santé liée à l'usage des produits de dépigmentation cutanée. Le coût médian pour l'approvisionnement mensuel des produits était de 1000 Francs CFA [600-1300]. Ces femmes s'approvisionnaient en produits de dépigmentation principalement dans les marchés (79,94%). Les femmes qui présentaient des pathologies cutanées et ayant un diagnostic préétabli représentaient 35,41% pour l'acné et 11,19% pour les tâches d'hyperpigmentation. Les complications médicales d'ordre esthétique qui étaient survenues après l'usage des produits de dépigmentation étaient l'acné (32,29%), les vergetures (13,44%), la dyschromie (14,10%), l'hyperpigmentation (9,14%), les dermatophyties (2,19%) et l'atrophie cutanée dans 1,24% des cas.

**Analyse univariée**: les femmes jeunes, ayant entre 15 et 34 ans étaient plus susceptibles d'utiliser les produits dépigmentant que les femmes qui avaient plus de 35 ans. Les élèves, étudiantes, ou apprenties utilisaient plus les produits dépigmentant que les femmes fonctionnaires, salariées, ouvrières ou artisanes (OR=3,83; IC95%=[1,16-12,63]). Les femmes qui n'étaient pas en couple (célibataires, veuves, séparées, divorcées) étaient plus susceptibles d'utiliser les produits de dépigmentation cutanée (OR=3,56; IC95%=[1,59-7,96]). Les femmes qui étaient à la recherche d'un partenaire utilisaient plus les produits de dépigmentation volontaire (PDV) que les autres (OR=5,52; IC95%=[1,19-25,48]. Les femmes qui n'appartenaient pas à une association ou à un groupe culturel, étaient plus susceptibles d'utiliser les PDV que celles qui étaient membre d'une association (OR=5,13; IC95%=[1,71-15,35]. Les femmes qui avaient un diagnostic préétabli de tâches d'hyperpigmentation, étaient plus susceptibles d'utiliser les PDV. La connaissance d'un effet néfaste pour la santé n'était pas associée à l'UPDV (Tableau 1, Tableau 1(suite)).

**Tableau 1:** Analyse uni variée des facteurs associés à l’usage des produits de dépigmentation volontaire chez les femmes de 15 à 49 ans de la zone sanitaire de Comè, 2016

Variables explicatives	UPDV			
	n	%	OR brut [IC_95 %_]	P-value
**Age**				**0,0050**
15-24 ans	201	87,11	3,1 [1,48-6,49]	
25-34 ans	186	73,74	1,28 [0,69-2,40]	
35-49 ans	124	68,53	1	
**Ethnie**				0,7531
Sahouè /Fon/ Aïzo	329	78,57	1	
Pédah/ Popo	85	80,7	1,13 [0,58-2,23]	
Yorouba/Nago	5	89,1	2,22 [0,22-22,14]	
Adja/ Mina	92	75,21	0,82 [0,40-1,70]	
**Religion**				0,5200
Christianisme	295	79,3	1	
Traditionnelle	159	73,3	0,71 [0,41-1,24]	
Athéisme	52	82,27	1,21 [0,49-2,97]	
Islam	5	77,94	0,92 [0,13-6,15]	
**Niveau d’étude**				0,4411
Primaire	161	82,05	1,29 [0,65-2,58]	
Secondaire/Supérieur	183	77,87	1	
Non scolarisée/alphabétisée	167	74,64	0,83[0,43-1,60]	
**Profession**				**0,0029**
Elève/Etudiante/Apprentie	144	91,39	3,83 [1,16-12,63]	
Agricultrice	44	61,11	0,56 [0,22-1,43]	
Fonctionnaire/salariée /ouvrière/artisanes	83	73,47	1	
Commerçantes/vendeuses/	187	78,03	1,28 [0,59-2,77]	
Ménagère/Sans profession	53	60,98	0,56 [0,21-1,46]	
**Milieu de résidence**				0,9604
Rural	357	78,19	1,01 [0,56-1,81]	
Urbain	154	77,94	1	
**Statut matrimonial**				**0,0012**
Mariée en Monogamie/Polygamie/ Concubinage	324	71,57	1	
Célibataire/Veuve/séparée/divorcée	187	89,98	3,56 [1,59-7,96]	

**Tableau 1(suite):** Analyse uni variée des facteurs associés à l’usage des produits de dépigmentation volontaire chez les femmes de 15 à 49 ans de la zone sanitaire de Comè, 2016

Variables explicatives	UPDV			
	n	%	OR brut [IC_95 %_]	P-value
**Motivation**				**0,0144**
Beauté	300	81,26	1,86 [1,05-3,28]	
Estime de soi	174	69,94	1	
Recherche de partenaire	37	92,78	5,52 [1,19-25,48]	
**Exigence de conjoint/partenaire**				0,7983
Oui	165	77,24	0,93 [0,53-1,61]	
Non	346	78,48	1	
**Influence ami, voisin, parent, proche**				0,1981
Oui	262	81,05	1,43 [0,82-2,49]	
Non	249	74,87	1	
**Niveau de bien-être économique**				0,1586
Pauvre	204	72,8	0,77 [0,39-1,51]	
Moyen	160	83,9	1,50 [0,71-3,17]	
Riche	147	77,54	1	
**Revenu mensuel (Fr CFA)**				0,9729
Moins de 10.000	278	78,49	1,07 [0,52-2,19]	
10.000-20.000	127	78,11	1,05 [0,48-2,27]	
20.000 et plus	106	77,20	1	
**Source supplémentaire d’argent**				0,1707
Conjoint/Partenaire/Parent/membre de la famille/ Ami	447	79,03	1,61 [0,80-3,23]	
Personnelle	64	69,97	1	
**Diagnostic préétabli d’acné**				0,0532
oui	149	86,52	2,05 [0,98-4,31]	
Non	362	74,06	1	
**Diagnostic préétabli de tâches D’hyperpigmentation**				**0,0002**
Oui	55	96,89	10,00 [2,29-43,63]	
Non	456	75,71	1	

Analyse multi-variée: de l'analyse multivariée, il ressortait que toutes choses étant égales par ailleurs, les femmes qui n'étaient pas en couple, les femmes qui étaient à la recherche de partenaires et celles qui présentaient des tâches d'hyperpigmentation cutanée étaient plus susceptibles d'utiliser les PDV avec des estimations respectives du risque par odds ratios ajustés de 3,1, 4,92 et 10,32 (Tableau 2).

**Tableau 2:** Analyse multi-variée des facteurs associés à l’usage des produits de dépigmentation cutanée chez les femmes de la zone sanitaire de Comè, 2016 (n = 511)

Variables explicatives	Usage des produits de dépigmentation cutanée		
	OR ajusté	IC_95 %_	P-value
**Statut matrimonial**			
Mariée en Monogamie/Polygamie/ Concubinage	1		
Célibataire/Veuve/séparée/divorcée	3,10	[1,29-7,44]	0,011
**Motivation**			
Beauté	1,09	[0,59-2,04]	0,768
Estime de soi	1		
Recherche de partenaire	4,92	[1,20-20,09]	0,021
**Diagnostic préétabli de tâches d’hyperpigmentation**			
Oui	10,32	[2,87-37,01]	0,000
Non	1		

## Discussion

**Synthèse des principaux résultats**: l'UPDV était prévalent chez 8 femmes sur 10. Les principes actifs des produits de dépigmentation utilisés par les femmes de la zone sanitaire de Comè étaient constitués majoritairement par l'hydroquinone, puis dans une faible proportion par les dermocorticoïdes. Une relation statistiquement significative a été retrouvée avec l'UPDV pour le statut matrimonial, la recherche d'un partenaire, et l'existence préétablie de tâches d'hyperpigmentation.

**Forces et limites de l'étude**: notre technique d'enquête a permis d'avoir un échantillon qui était représentatif de la population des femmes en âge de procréer de la zone sanitaire de Comè. L'inventaire des produits cosmétiques cutanés utilisés par chaque femme, ainsi que l'analyse de la liste des actifs entrant dans la composition de chaque produit a permis de d'identifier les actifs dépigmentants connus de la revue de littérature. Les limites de la présente étude résident dans la non prise en compte éventuelle d'actifs dépigmentants non encore documentés dans la littérature et dans la non prise en compte d'autres modes systémiques de dépigmentation cutanée tels que les injections ou l'absorption de substances médicamenteuses.

**Discussion des principales différences**: une prévalence inférieure de l'UPDV a été retrouvée dans une étude réalisée par Nguenmegne dans la ville de Parakou qui était de 72,10% [18]. Cette différence qui n'est pas très importante pourrait s'expliquer par le caractère très touristique de Comè qui est par ailleurs une ville côtière avec beaucoup d'échanges marchands sur le corridor Abidjan Lagos [2]. Au Soudan, Abubakar *et al* ont retrouvé une prévalence d'UPDV de 79,60%. Cette prévalence était proche de celle de notre étude, cependant cette prévalence a été obtenue chez les étudiantes sélectionnées dans les universités du centre du Soudan et non chez des femmes retrouvées dans la population générale. En Afrique, plusieurs études [9-11] ont trouvé des prévalences d'UPDV comprise entre 25 et 96%. A Lomé au Togo, Pitchet *et al* [21] ont trouvé une prévalence de 58,9% qui est inférieure à la nôtre. Cette différence pourrait être expliquée par la différence dans la méthodologie entre les deux études. En effet la sélection des cibles était non probabiliste avec une technique d'échantillonnage par choix raisonné. Les enquêtes se sont déroulées au grand marché de Lomé, au Campus Universitaire, dans un collège d'enseignement général et dans les bureaux de la poste centrale de la ville. En Thaïlande, Peltzer *et al* [22] ont trouvé une prévalence égale à 89,1% chez les étudiantes. Cette prévalence est largement supérieure à la nôtre. Cette différence pourrait s'expliquer par la différence entre les méthodes d'études d'une part et par le niveau socioéconomique des cibles et celui général des populations qui diffère entre les deux pays. En effet, cette étude s'est déroulée dans les universités des grandes villes. Dans la recherche des prévalences, plusieurs auteurs ont trouvé que les principaux produits de dépigmentation cutanée utilisés étaient à base d'hydroquinone et des dermocorticoïdes: Kamagaju *et al* à Kigali au Rwanda [23], Kebe *et al* en Mauritanie [24], Mugisho *et al* [4], Hamed *et al* en Jordanie [2]. Les produits dépigmentants étaient facilement accessibles aux femmes à cause de leur prix relativement bas. Ces résultats ont été aussi trouvés par Kamagaju et al à Kigali [23]. Hamed et al en Jordanie, avaient trouvé que le coût moyen mensuel des produits de dépigmentation était de 28USD (16240 Francs CFA) [2]. Ce coût mensuel des produits était supérieur au coût mensuel retrouvé dans notre étude. Cette différence pourrait s'expliquer par le fait qu'en Jordanie, les produits cosmétiques étaient très contrôlés et feraient l'objet des taxes, ce qui ferait élever leur prix. Les femmes célibataires, veuves, séparées ou divorcées faisaient plus usage des produits de dépigmentation cutanée que celles qui étaient en couple. Ces femmes n'étant pas en couple, pour se faire belles et désirables avaient probablement recours aux produits dépigmentant de la peau [25]. Cette association a été trouvée aussi dans l'étude de Fouda au Cameroun [26]. L'utilisation des produits dépigmentants à Lomé était plus fréquente chez les célibataires avec une proportion de 82,9 % par rapport aux mariées (43,3%) [21]. La motivation pour faire usage des produits de dépigmentation cutanée varie d'une femme à l'autre. Les résultats de la présente étude ont montré que le risque de faire usage des produits de dépigmentation chez les femmes à la recherche de partenaire ajusté à d'autres facteurs, était de 4,92 fois plus élevé, comparées aux femmes qui recherchaient l'estime de soi. L'association entre la recherche de partenaire et l'usage des produits de dépigmentation cutanée a été trouvée aussi par une étude réalisée par Hamed *et al* à Amman en Jordanie [27]. Dans cette dernière étude, les femmes avaient également approuvé que l'usage des produits de dépigmentation favoriserait l'estime de soi, la perception de la beauté et de la jeunesse, le mariage et les possibilités d'emploi par rapport aux non-utilisatrices. L'existence de diagnostic préétabli de tâches d'hyperpigmentation cutanée justifie le plus souvent une prescription médicale, ce qui explique la relation notée dans la présente étude entre l'UPDV et ce facteur. Ainsi, les principales motivations à l'UPDV dans la zone sanitaire de Comè sont d'ordre surtout social et l'UPDV n'est pas associé à la connaissance des effets secondaires néfastes pour la santé de cette pratique. L'identification des stratégies idoines pour la réduction de cette pratique devra nécessairement tenir compte de ces spécificités.

## Conclusion

Cette étude a permis de déterminer la prévalence de l'usage des produits de dépigmentation cutanée dans la zone sanitaire de Comè en 2016. Cette prévalence était parmi les plus élevées rapportées pour l'heure en Afrique de l'Ouest. Les facteurs associés à l'usage des produits de dépigmentation cutanée étaient le statut matrimonial, la recherche de partenaires, et l'existence préétablie de tâches d'hyperpigmentation cutanée. Pour lutter contre le phénomène ainsi observé, des interventions doivent être orientées prioritairement vers la communication pour le changement de comportement, la mobilisation sociale et le renforcement des capacités des prestataires de santé en tenant compte du contexte. Un accompagnement par la mise en place d'un cadre juridique et législatif est indispensable afin d'avoir des outils efficaces de contrôle de la commercialisation des produits de dépigmentation.

### Etat des connaissances actuelles sur le sujet

- L'usage des produits de dépigmentation volontaire est une pratique courante chez les femmes de tous les continents;
- L'hydroquinone est le produit le plus fréquemment utilisé.

### Contribution de notre étude à la connaissance

- L'usage des produits de dépigmentation volontaire est courant chez 8 femmes sur 10 dans la zone sanitaire de Comè;
- Les facteurs de motivation sont essentiellement d'ordre social et sont relatifs au statut matrimonial et à la recherche de partenaire;
- La connaissance d'effets sanitaires néfastes ne semble pas influencer la pratique d'usage des produits de dépigmentation volontaire.

## Conflits d’intérêts

Les auteurs ne déclarent aucun conflit d’intérêts.
